# Regulation of bone mineral density in the grey squirrel, *Sciurus carolinensis:* Bioavailability of calcium oxalate, and implications for bark stripping

**DOI:** 10.1111/jpn.12740

**Published:** 2017-06-12

**Authors:** C. P. Nichols, N. G. Gregory, N. Goode, R. M. A. Gill, J. A. Drewe

**Affiliations:** ^1^ Royal Veterinary College London UK; ^2^ Forest Research Farnham Surrey UK

**Keywords:** CaOx, low‐calcium diet, micro‐CT, pQCT, pyridinoline, tree damage

## Abstract

The damage caused when grey squirrels strip the outer bark off trees and ingest the underlying phloem can result in reduced timber quality or tree death. This is extremely costly to the UK forestry industry and can alter woodland composition, hampering conservation efforts. The calcium hypothesis proposes that grey squirrels ingest phloem to ameliorate a seasonal calcium deficiency. Calcium in the phloem predominantly takes the form of calcium oxalate (CaOx), however not all mammals can utilise CaOx as a source of calcium. Here, we present the results of a small‐scale study to determine the extent to which grey squirrels can utilise CaOx. One of three custom‐made diets containing calcium in varying forms and quantities (CaOx diet, Low‐calcium carbonate (CaCO_3_) diet and Control diet) were fed to three treatment groups of six squirrels for 8 weeks. Bone densitometric properties were measured at the end of this time using peripheral quantitative computed tomography and micro‐computed tomography. Pyridinoline—a serum marker of bone resorption—was measured regularly throughout the study. Bone mineral density and cortical mineralisation were lower in squirrels fed the CaOx diet compared to the Control group but similar to that of those on the Low‐calcium diet, suggesting that calcium from calcium oxalate was not effectively utilised to maintain bone mineralisation. Whilst no differences were observed in serum pyridinoline levels between individuals on different diets, females had on average higher levels than males throughout the study. Future work should seek to determine if this apparent lack of ability to utilise CaOx is common to a large sample of grey squirrels and if so, whether it is consistent across all areas and seasons.

## 1 INTRODUCTION

Damage caused by the grey squirrel, *Sciurus carolinensis*, to trees in the UK can impose a vast economic toll on UK forestry due to deterioration of timber quality (Mayle & Broome, 2013). Grey squirrels strip off the outer bark to ingest the underlying phloem, and severe damage can pre‐maturely kill younger trees (Mountford, Peterken, Edwards, & Manners, 1999). This can result in a change in woodland composition making the conservation of culturally and biologically important sites difficult in the UK (Mountford, 1997). The grey squirrel was introduced to Great Britain in the late 19th century (Middleton, 1930) and to Ireland in the early 20th century and has since been released in Italy (Lurz et al., 2001), posing a threat to both the native red squirrel, *Sciurus vulgaris*, and to vulnerable woodland (Signorile & Evans, 2007).

Many studies have attempted to determine what triggers the grey squirrel to damage trees (Kenward & Parish, 1986; Kenward et al., 1996), with a view to informing preventive approaches; however, the underlying causal mechanism is still unknown. It has been suggested that bark stripping represents an attempt to utilise inner bark as a source of carbohydrate when other food resources are lacking (Kenward, 1983; Kenward, Parish, Holm, & Harris, 1988; MacKinnon, 1976); however, bark stripping still occurs in semi‐wild enclosures when food is provided ad libitum, and bark is of low calorific value (Kenward, 1982). In some cases, bark stripping may be the result of agonistic behaviour between individuals, for instance young males are often implicated (Taylor, 1966, 1969). This does not however preclude other drivers for bark stripping such as the seeking of a trace nutrient that may be deficient (Allen, 1943).

The calcium hypothesis proposes that grey squirrels ingest phloem to ameliorate a seasonal calcium deficiency (Nichols, Drewe, Gill, Goode, & Gregory, 2016). Calcium is found in greater quantities than any other inorganic element in plants (McLaughlin & Wimmer, 1999). It is present in the phloem of tree bark in large quantities and precipitates as calcium oxalate (CaOx) crystals in the cell vacuoles of many species of angiosperm trees (Borchert, 1990), acting as a store as excess calcium is sequestered (Franceschi & Nakata, 2005; Hudgins, Krekling, & Franceschi, 2003). These crystals can occur in most plant families, including oak, *Quercus robur* and poplar, *Populus tremula* (Trockenbrodt, 1995), both of which are known to be susceptible to damage by the grey squirrel (Rowe & Gill, 1985). It is possible that juveniles and pregnant adult females of some grey squirrel populations have an increased demand for dietary calcium during the bark‐stripping season (Nichols, 2016) of April‐July (Gurnell, 1987; Shorten, 1957).

If, as the calcium hypothesis suggests, grey squirrels are damaging trees to ingest calcium, it would be expected that grey squirrels can utilise CaOx. However, not all mammals can utilise CaOx because it is poorly absorbed unless it is broken down into its constituent parts (Hossain, Ogawa, Morozumi, Hokama, & Sugaya, 2003). The resultant oxalate proves problematic as it is a dietary deterrent for grey squirrels (Schmidt, Brown, & Morgan, 1998), and oxalic acid can be poisonous to mammals (Blackwell, 1990), unless it is degraded. This breakdown can be achieved by some mammals using symbiotic microbes in the gut such as *Oxalobacter* species (Palgi, Taleisnik, & Pinshow, 2008). The pack rat, *Neotoma albigula* **,** and the fat sand rat, *Psammomys obesus* **,** for instance can achieve this feat (Shirley & Schmidt‐Nielsen, 1967), and it is possible that grey squirrels could also. The CaOx complex is practically inert (Hossain et al., 2003), and so if grey squirrels lack the ability to break it down, it will likely pass through the body and be excreted unchanged, with no calcium absorbed.

Bone is a dynamic tissue that is constantly being remodelled by the antagonistically coupled actions of osteoblasts depositing, and osteoclasts resorbing, bone. The synergy of these cells can become uncoupled in mammals in response to various stressors such as changes in diet (Demigne et al., 2006; Gennari, 2001; Rodriguez‐Martinez & Garcia‐Cohen, 2002), resulting in more bone being resorbed than deposited, and decreased bone mineral density (BMD). Maintaining the skeleton at a desired adequate mineral density and content depends on, amongst other factors, the quantity of calcium ingested (Heaney, 2001). Therefore, individuals maintained on a diet in which the main source of calcium is CaOx will likely undergo increased bone resorption and reduced BMD if calcium is not being absorbed, when compared with individuals on a normal skeletal maintenance diet. Measuring bone parameters after prolonged exposure to treatment diet are a reliable way of estimating the long‐term effects of different calcium intakes in humans and rats (Guéguen & Pointillart, 2000).

The aim of this study was to determine the extent to which grey squirrels can utilise CaOx by feeding custom‐made diets containing varying levels of calcium in different forms: calcium carbonate (CaCO_3_), calcium from which is efficiently absorbed in the intestine and retained for bone mineralisation in mammals (Matkovic, Fontana, Tominac, Goel, & Chesnut, 1990; Prince et al., 1995; Smith, Gilligan, Smith, & Sempos, 1989), and CaOx—the bioavailability of which is unknown in grey squirrels. If one group of grey squirrels is fed a diet low in CaCO_3_, and another group fed a diet containing adequate levels of CaCO_3_, the BMD and levels of bone resorption would be expected to differ accordingly. These two groups would then act as opposites of potential outcome for comparison with a third group, fed a diet in which the primary source of calcium is CaOx, indicating the extent to which it can be utilised by grey squirrels.

## 2 MATERIALS AND METHODS

### 2.1 Study design

Squirrels were trapped and handled according to guidelines approved by Forest Research and the Royal Veterinary College—University of London ethical review committees. All housing and procedures were carried out in an establishment licensed under the Animal (Scientific Procedures) Act 1986, with a project licence, and by individuals holding personal licences issued by the UK Home Office. Eighteen grey squirrels were trapped in Alice Holt Forest, Hampshire, comprising 10 males (all adults), and eight females (seven adults and one subadult). This was an arbitrary sample size chosen for this small‐scale study to garner an understanding of the effect of the three custom‐made treatment diets, and because it is divisible by three. This number was also restricted by the cost of the treatment diets, and an ethical obligation to keep the total number of individuals involved to no more than scientifically necessary. Age was determined by examining the extent of epiphyseal fusion by radiograph (Dubock, 1979). Squirrels were housed individually with 12 hr of light and darkness, and regulated ambient temperature. Cages consisted of a nest box with a piece of hessian sack for bedding, 3D climbing enrichment and a gnaw block. Squirrels had access to an enrichment cage with a forage tray and additional 3D climbing apparatus 50% of the time. All squirrels had deionised water freely available and were initially fed a diet of equal parts peanuts, wholegrain wheat, maize and sunflower seeds during acclimatisation, before being weaned on to the pelleted control diet. The duration of weaning varied between individuals, taking approximately 1 week. Individuals were weaned to facilitate a smooth transition on to the treatment diets.

The animals were then randomised within sexes using the stratified weight method to one of: control diet (0.9% CaCO_3_), low‐Ca diet (0.1% CaCO_3_) or CaOx diet (0.1% CaCO_3_ + 2.5% CaOx). The custom‐made diets were fed for 8 weeks and were obtained from Special Diet Services, Witham, UK. All animals were fed 30 g/day of the allocated diet. Uneaten food was weighed daily to monitor food consumption, and squirrels were weighed weekly to monitor condition. A monitoring sheet was designed to monitor the progress and welfare of each individual, on which daily food ingestion was recorded, weekly weight measurements and miscellaneous notes pertaining to welfare. In addition, a distress score was devised after Wolfensohn and Lloyd (2013), which was performed after each squirrel was weighed. Blood samples were taken every 2 weeks, as this was deemed an appropriate interval to take the 1.5 ml of blood necessary for the study, based on the grey squirrels body size and total blood volume. The tail‐bleeding technique was used, with EMLA anaesthetic cream to reduce pain caused by the minor scalpel incision, and sealed with veterinary glue to facilitate wound closure, in a separate animal laboratory to the animal housing room. Serum was stored at −80°C. All individuals were euthanised using carbon dioxide at the endpoint of the study.

Immediately after death individuals were dissected and body condition recorded on two‐five‐point scales, one of subcutaneous fat and one of peritoneal fat. Kidneys and bladders were dissected out for microscopic analysis for the presence of calculi. The right femur was dissected out and the length measured using callipers, wrapped in phosphate‐buffered saline‐soaked gauze and cling‐film, before being frozen at −20°C for later bone densitometric analysis. Blood samples were taken after death via cardiac puncture and serum stored at −80°C for later analysis of bone resorption marker.

### 2.2 Bone resorption marker

Serum levels of pyridinoline (PYD) were measured regularly using a MicroVue^™^serum PYD EIA kit (Quidel Corporation, San Diego, USA). Crosslinks such as PYD form between collagen molecules in bone (Knott & Bailey, 1998), and as bone is degraded during resorption, PYD is released into the blood. Increased serum PYD levels therefore indicate increased bone resorption. Data were normalised based on the average of an internal control for each assay kit used.

### 2.3 pQCT analysis

Total bone mineral density was measured using peripheral quantitative computed tomography (pQCT) with an XCT2000 machine (Norland Stratec Medizintechnik GmbH, Birkenfeld, Germany). Quality assurance measurements were made prior to measurements using cone and standard phantoms. Femurs were placed condyles facing down in the pQCT gantry. The parameters of operation were contour mode one, peel mode two, threshold 180 mg/cm^3^, resolution 70 μm. A guide beam was aligned at the distal tip of the femur, and seven points scanned: 2.5% from the distal tip, and five further scans in 0.75 mm increments proximally, and at 50% of femur length. The distal metaphysis of the femur was chosen as the section of interest because, after preliminary scans on the proximal and distal metaphysis of both squirrel tibia and femur, the most meaningful quantity of trabecular bone was found here. Trabecular bone is rapidly remodelled in response to load or nutritional changes, acting as an early and accurate indicator of bone metabolic activity (Geng, DeMoss, & Wright, 2000; Kalu & Orhii, 1999; Seto, Aoki, Kasugai, & Ohya, 1999; Teofilo, Azevedo, Petenusci, Mazaro, & Lamano‐Carvalho, 2003; Zheng et al., 2007).

### 2.4 μCT analysis

Femurs were scanned using a Skyscan 1172 micro‐computed tomography (μCT) system (Bruker microCT, Kontich, Belgium). Scans were obtained using an 8 μm resolution, and 0.5 mm aluminium filter. Images were reconstructed using NRecon (Bruker microCT, Kontich, Belgium). The region of interest for bone architecture properties to be measured using Bruker CT‐Analyser Version 1.13 (Bruker microCT, Kontich, Belgium) was selected so as to replicate the location of pQCT scans.

### 2.5 Statistical analysis

Statistical analysis was performed in R version 3.1.2 (R Core Team 2014). Multiple regression and ANCOVA were used to analyse BMD data, and PYD data were analysed using a mixed effects model to take into account temporal pseudo‐replication. The effects of treatment diet, sex and femur morphometry were analysed, controlling for body mass and body condition.

## 3 RESULTS

No morphometric differences in grey squirrel femurs were observed between treatment groups in terms of femur length (*p* = .667, *df* = 15), mid‐point width (*p* = .367, *df *= 15) and depth (*p* = .135, *df* = 15).

### 3.1 Bone mineral density

**Figure 1:**
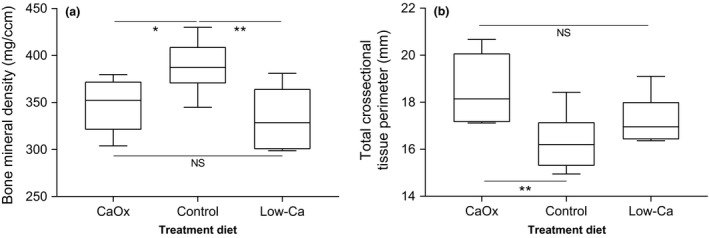
Significant differences in distal femoral bone densitometric properties between cohorts of grey squirrels fed one of three treatment diets: CaOx diet (*n* = 6), Control diet (*n* = 6) or Low‐Ca diet (*n* = 6). (a) Total bone mineral density measured by pQCT (*p* = .0182, *df* = 15); (b) Mean total cortical cross‐sectional tissue perimeter measured by μCT. NS – *p *=> .05, *p =< .05, **p = <.005

The total femoral BMD of grey squirrels varied depending on the treatment diet fed throughout the study (*p* = .0182, *df* = 15) (see Figure 1a). Squirrels fed the Control diet had a higher BMD than those on the CaOx diet (*p* = .0355) and the low‐Ca diet (*p* = .00672), but no difference was observed between the BMD of squirrels on the CaOx diet and the low‐Ca diet (*p* = .4191). No differences in BMD were observed between the sexes (*p* = .669, *df* = 16), and there was no statistical interaction between the variables treatment diet and sex in determining BMD (*p* = .8323, *df* = 12).

### 3.2 Bone architecture

Utilising μCT produces a large number of densitometric variables. Significant differences were observed between the femurs of squirrels depending on treatment diet in some metrics, for instance the mean total cortical cross‐sectional tissue perimeter, in which the CaOx diet cohort had a longer tissue perimeter than the control diet (*p* = .0085, *df* = 15), but was not different from those fed the low‐Ca diet (*p* = .0996) (see Figure 1b). Other metrics such as trabecular bone volume did not show differences depending on treatment diet (*p* = .73, *df* = 15), but instead varied with sex. Females had increased trabecular bone volume compared with males (*p* = .0291, *df* = 16).

### 3.3 Pyridinoline

**Figure 2:**
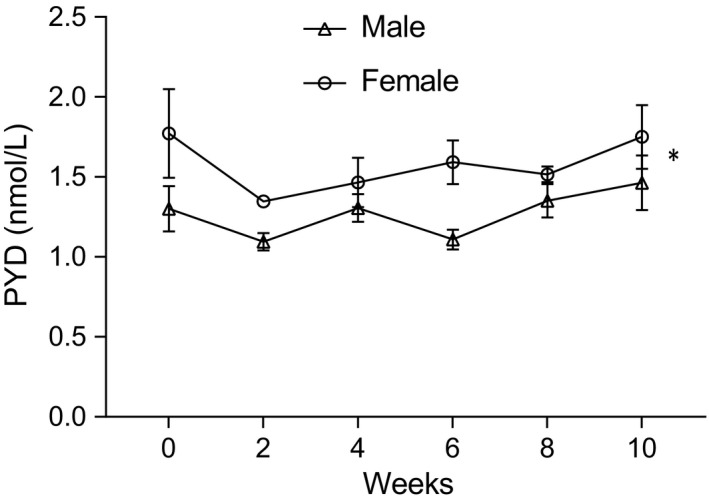
Average serum pyridinoline (PYD) levels in grey squirrels were significantly higher in females than males over the 10 weeks of the study. (Female *n* = 8, male *n* = 10). Bars indicate standard error of the mean. NS – *p* => .05, *p  =  <.05

No significant effect was observed of treatment diet fed throughout the study on the concentration of serum PYD over time, and no interaction between treatment diet and sex was found. However, females had higher levels of serum PYD than males (*p* = .0292, *df* = 16; see Figure 2).

### 3.4 Microscopy

Upon dissection of the kidney and bladder, one male and one female squirrel on the CaOx diet were found to have small calculi, measuring approximately 1 mm across.

## 4 DISCUSSION

This small‐scale study of calcium utilisation in grey squirrels has produced several key results from which inferences can be made. It appears that grey squirrels may not be able to utilise CaOx as a source of calcium, as the femoral total BMD of squirrels fed the CaOx diet did not differ significantly from that of squirrels fed the low‐Ca diet. It did however differ significantly from squirrels on the control diet. Low‐calcium diets are commonly used to model bone loss for osteoporosis‐related studies in rats (Salomon, 1972; Zhang, Dong, Leung, & Wong, 2009), and in previous investigations, the level of calcium used in the present study—0.1%, have been shown to alter the bones of rats over similar timescales (Jiang et al., 2004; Zhang et al., 2009). The squirrels on the CaOx diet ingested 18 times the quantity of calcium by mass than squirrels on the low‐Ca diet, and yet there was no significant difference in BMD. Higher levels of dietary calcium would be expected to have the effect of increasing BMD, as can occur in humans (Recker & Heaney, 1985), if calcium is in a utilisable form. Squirrels on the control diet however had significantly higher BMD compared with squirrels on the low‐Ca diet, whilst ingesting only nine times the quantity of calcium by mass. As the vast majority of calcium present in the CaOx diet was CaOx, it is likely the reason it did not get incorporated in the bone turnover process to replace bone lost through resorption is due to grey squirrels being unable to utilise the CaOx complex to a significant extent.

This finding is corroborated by the μCT results, as the femoral mean total cortical cross‐sectional tissue perimeter of squirrels on the CaOx diet was significantly longer than squirrels on the control diet. There was no difference in femoral length or width, so this likely indicates the cortical tissue perimeter has thinned, as has been shown in mice (Lim et al., 2015). This means less cortical bone was present in the femurs of squirrels on the CaOx than those on the control diet. A reduction in cortical bone has been linked to a diet deficient in calcium in rats (Shen, Birchman, Xu, Lindsay, & Dempster, 1995), and so a reduction in grey squirrels suggests an inability to utilise CaOx. Whilst not all bone properties measured by μCT showed significant differences between the treatment diet groups, some that were insignificant such as trabecular bone volume, tended to have the same pattern as was seen in the pQCT results. The bones of individuals on the CaOx diet were more similar to those on the low‐Ca diet than the control diet, as would be expected if CaOx was not being utilised. This lack of significance is likely due to the small sample size.

Unexpectedly, concentrations of serum PYD—a marker of bone resorption (Seibel, Robins, & Bilezikian, 1992)—did not vary between treatment groups. If CaOx is not being utilised, it might be expected that bone resorption would increase due to inadequate dietary calcium, as has been shown in mice fed such a diet (Zheng et al., 2007). It would however also be expected that bone resorption would increase in squirrels fed the low‐calcium diet, yet this was not evident from the PYD results. Levels of PYD can be highly variable both between and within individual humans (Ginty, Flynn, & Cashman, 1998), which is likely to also be the case in other mammals. The variation in serum PYD between individuals and over time may have been too great, and sample size too small, to distinguish significant differences. Alternatively, bone resorption, and therefore PYD (Seibel et al., 1992), may have remained relatively constant throughout the study, whilst levels of bone formation dropped, uncoupling bone turnover. Measuring a marker of bone formation for comparison with PYD would be beneficial for any future work building on this small‐scale study (Eastell et al., 1993); however, reagents and kits are not routinely standardised for use in squirrels, and lack of cross‐reactivity excluded the use of most markers in preliminary experiments.

There were however significant differences in the concentration of serum PYD between the sexes, with females showing higher levels of bone resorption than males. Sex‐specific differences in bone turnover have been identified previously in rats based on diet (Zengin et al., 2015). However, this was based on a low‐carbohydrate, high‐fat diet, so it is unlikely grey squirrels are showing a differential response to the custom‐made diets. Sex differences were also identified in rats in response to simulated weightlessness (Hefferan et al., 2003), which can be thought of as a special kind of disuse osteoporosis (Milstead, Simske, & Bateman, 2004). Variable bone turnover in males and females led to differences in bone architecture in response to hind‐leg unloading (Hefferan et al., 2003). Whilst the squirrels were not subjected to this level of immobilisation, and they were restricted to an individual cage, which resulted in reduced activity compared to that which would normally occur in the wild (Gurnell, 1987). Indeed the BMD of captive squirrels was generally lower than that of wild grey squirrels (Nichols, 2016), likely due to this reason, and the negative effect reduced weight‐bearing can have on BMD. The difference in PYD levels between males and females may reflect a similar sex‐specific response to reduced loading as in rats, because some bone architectural differences were also observed between males and females. For instance, trabecular bone volume was higher in female grey squirrels compared with males.

Ingesting large quantities of oxalate can increase the risk of forming calculi in the kidney and bladder in humans for instance (Williams & Wandzilak, 1989); however, this is due to oxalate chelating calcium in the plasma to form CaOx after absorption has taken place through the gut, leading to muscle disorders and deposition of CaOx crystals in tissues (De Lorenzi, Bernardini, & Pumarola, 2005; Knoll et al., 2004; Marangella, Vitale, & Petrarulo, 1996; Petrarulo, Vitale, Facchini, & Marangella, 1998). In the present study, the CaOx complex was ingested whole, as would be the case when grey squirrels ingest tree phloem (Franceschi & Nakata, 2005). The CaOx complex is practically physiologically inert and would not usually be absorbed (Shirley & Schmidt‐Nielsen, 1967). As squirrels on the CaOx diet were observed to have bladder calculi, this could indicate that the calcium oxalate complex was being broken down at least to some extent, for instance by oxalate‐degrading bacteria in the gut before absorption (Allison, Cook, Milne, Gallagher, & Clayman, 1986), and the stones forming due to the resultant oxalate in the plasma accumulating in the bladder (Williams & Wandzilak, 1989). However, intestinal absorption does not necessarily reflect the bioavailability of calcium to be used in bone mineralisation (Guéguen & Pointillart, 2000), as calcium may still not be available to prevent bone loss (Cashman, 2002), as the oxalate would chelate calcium at the same ratio as the CaOx ingested, resulting in no net calcium gain. The CaOx complex may also have been absorbed whole, as has been shown in rats (Hanes, Weaver, Heaney, & Wastney, 1999).

Those mammals that are able to utilise CaOx as a source of calcium, such as some rats, rabbits (*Oryctolagus cuniculus*), guinea pigs (*Cavia porcellus*), swine (*Sus scrofa*), horses (*Equus caballus*) and sheep (*Ovis aries*) do not necessarily have the ability to utilise it consistently throughout the year or their lifetime, as it can be induced and regulated by the levels of oxalate in the diet (Allison & Cook, 1981; Allison, Littledike, & James, 1977). Whilst the present results do not point to adult grey squirrels being able to utilise CaOx, it may be that they can induce a seasonal ability to do so, or can do so at certain life stages, for instance by increasing the dietary oxalate when ingesting phloem. As this study did not take place during the bark‐stripping season of April‐July, we cannot say that grey squirrels cannot utilise CaOx during the bark‐stripping season. Also as no juveniles were used during the study, we cannot say that juveniles cannot utilise CaOx. The same is true of pregnant or lactating female squirrels. Also, when a substance such as calcium is being craved, the substance in question does not even need to satisfy the deficiency to be sought out (Olynyk & Sharpe, 1982). This suggests that even if grey squirrels cannot utilise calcium oxalate as a source of calcium, and they could still be stripping the bark off trees in an attempt to obtain calcium.

As this was a small‐scale study, future work should aim to determine if this apparent lack of ability to utilise CaOx is common to a larger sample of grey squirrels, and if it is consistent across all areas and seasons, before the calcium hypothesis is discounted. One way to do this would be to build on this study's foundation using radiolabelled CaOx to definitively determine if it is being incorporated into the bone matrix and therefore being utilised, and also to use metabolic cages to accurately record intake and excrement of calcium (Guéguen & Pointillart, 2000). Metabolic cages facilitate the collection of all urine and faeces produced by an individual, the analysis of which would indicate the extent to which CaOx passes through the animal unutilised. Also, whilst mineral blocks have been previously trialled as a delivery method to provide grey squirrels with trace nutrients that may be lacking without success (Tee, 1984), this study did not include calcium. A controlled trial in which a calcium block is provided in an attempt to prevent bark stripping may therefore be beneficial in testing the extent to which the calcium hypothesis has practical application.

In addition to the continuation of this approach examining the role of calcium in bark‐stripping behaviour, alternative hypotheses should also be investigated. For instance that grey squirrels are stripping bark due to a fondness for sugar. This theory has traditionally not been given much credence (Kenward et al., 1996); however, recent work (Nichols, 2016) suggests it may be time to re‐evaluate this. The present study is a novel and important step in investigating the motivations for grey squirrels to strip bark off trees. As understanding of the physiology behind this behaviour, and the nutritional limits that may govern it continues to increase, so do the chances of an effective preventive measure being developed.

## CONFLICT OF INTEREST

The authors state no conflict of interest.

## AUTHORS’ CONTRIBUTIONS

CPN conducted the fieldwork, laboratory work, analysis and drafted the manuscript; the other authors provided editorial, practical and supervisorial advice. All co‐authors approved the manuscript.
